# Addressing the data guardian and geospatial scientist collaborator dilemma: how to share health records for spatial analysis while maintaining patient confidentiality

**DOI:** 10.1186/s12942-019-0194-8

**Published:** 2019-12-21

**Authors:** Jayakrishnan Ajayakumar, Andrew J. Curtis, Jacqueline Curtis

**Affiliations:** Department of Population and Quantitative Health Sciences, School of Medicine, Case Western Reserve University, Cleveland, OH USA

## Abstract

**Background:**

The utility of being able to spatially analyze health care data in near-real time is a growing need. However, this potential is often limited by the level of in-house geospatial expertise. One solution is to form collaborative partnerships between the health and geoscience sectors. A challenge in achieving this is how to share data outside of a host institution’s protection protocols without violating patient confidentiality, and while still maintaining locational geographic integrity. Geomasking techniques have been previously championed as a solution, though these still largely remain an unavailable option to institutions with limited geospatial expertise. This paper elaborates on the design, implementation, and testing of a new geomasking tool *Privy*, which is designed to be a simple yet efficient mechanism for health practitioners to share health data with geospatial scientists while maintaining an acceptable level of confidentiality. The basic premise of Privy is to move the important coordinates to a different geography, perform the analysis, and then return the resulting hotspot outputs to the original landscape.

**Results:**

We show that by transporting coordinates through a combination of random translations and rotations, Privy is able to preserve location connectivity among spatial point data. Our experiments with typical analytical scenarios including spatial point pattern analysis and density analysis shows that, along with protecting spatial privacy, Privy maintains the spatial integrity of data which reduces information loss created due to data augmentation.

**Conclusion:**

The results from this study suggests that along with developing new mathematical techniques to augment geospatial health data for preserving confidentiality, simple yet efficient software solutions can be developed to enable collaborative research among custodians of medical and health data records and GIS experts. We have achieved this by developing *Privy*, a tool which is already being used in real-world situations to address the spatial confidentiality dilemma.

## Introduction

The following scenario is an all-too-common problem faced in the health care and delivery sector. A hospital emergency response manager, basically a doctor overseeing all paramedic call outs, wants a spatial analysis of all trips responding to an asthma exacerbation. The analysis should include location, times, subject (for example children) and medication given (an indication of severity). To perform a spatial hotspot analysis of these data, require geographic information system (GIS) skills the hospital doesn’t possess. Bringing in a spatial science collaborator would require Institutional Review Board (IRB) approval, and possibly the need for the analysis to occur within a secure onsite data environment. While having such safeguards is important, the sacrifice is the time it takes to have the hospital IRB approve the study, and the impediment of geographic distance if the collaborator has to perform the analysis on site. The solution we present in this paper is a direct result of this scenario, and many other situations where a health-geospatial collaboration is needed, often in near-real time, and no easy spatial confidentiality solution exists. While there has been considerable attention to various aspects of the spatial confidentiality problem, many involving elegant and powerful solutions, here we focus on utility and practicality.

A spatial appreciation continues to grow within the health sector, ranging from the addition of geographic locations in research to needs assessments (e.g., Health Impact Assessments, Community Health Improvement Plans, etc.), to spatially guided precision medicine [14]. Common requests between clinicians and researchers include tasks such as mapping patient locations, or finding distances between cases and the nearest clinic. Yet even though basic “map making” has become more ubiquitous, using either a geographic information system (GIS) or Google Earth [9, 11], these simple tasks still remain logistically challenging for many in the health profession, especially in terms of satisfying IRB protocols. Even more challenging is the ability to conduct more sophisticated spatial analysis [16, 26] where both implementation or a correct interpretation of the output is beyond many without a geoscience training [5]. For many health institutions, the in-house geospatial expertise for performing advanced techniques such as spatial clustering or exploratory spatial data analysis (ESDA) is limited [18]. Therefore, while a county health department may see the benefit of creating fine scale maps of opioid overdoses [48] or a children’s hospital might wish to understand its neighborhood child injury risk pattern [43] by overlaying hotspots onto built environment surveys, these tasks often remain unachievable due to a lack of geospatial skills [18]. While collaborations are possible with IRB oversight, the time taken to obtain such permission often takes considerable time. One solution is a collaborative partnership that could mine the ever expanding data, for example electronic medical records [40] if personal identifiers and precise spatial locations can be removed, while not impacting the ability for analysis. The demand for such a solution would be high as the utility would be broad, including disease mapping and analysis, health risk surveillance [8], outbreak response [19], healthcare delivery studies [20], identifying sub-neighborhood level health patterns [24], and clinical support.

Concern regarding spatial privacy and confidentiality [3, 10, 22], especially with regards health data [46] is justifiable. Arguably, the confidentiality conversation can be thought of in two ways; “in-house” map making where a “mapper” has access to data but through cartography reveals locations that can be re-engineered to an unacceptably precise level, and secondly, the ability to share data “out-of-house” to allow for expert analysis even though the mapping team is not allowed access to confidential records. These two problems are linked, because violations of inappropriate cartography leading to reengineering risk could occur either by the institution, or the out-of-house collaborator. Previous research on spatial privacy and spatial data re-engineering have revealed the severity of this problem using re-engineering examples. Curtis et al. [22], were able to identify mortality locations in the real world from published maps with only limited geographic features and boundaries through digitally scanning, geo-referencing, and digitizing before uploading the resulting coordinates into a GPS unit. Similarly, Brownstein et al. [13] used reverse geocoding and geo-referencing techniques to identify patient locations from a prototypical map of randomly selected patients. They were able to successfully identify 26%, 51.6%, 70.7%, and 93% of addresses within one, five, ten and twenty buildings. Further, they extended the results to create an unsupervised learning algorithm [12] that could automatically classify patient location with an accuracy of 79%, revealing the vulnerability of point maps. At a broader scale, Kounadi and Leitner [34] found that over an eight-year period, more than 68,000 home addresses were made vulnerable from a set of forty-one academic articles. Worryingly, their study revealed that at the time of writing that this risk remained an ongoing problem in academically published maps. It is therefore understandable that an IRB, while more traditionally experienced with health record protection, should consider the vulnerabilities of the spatial dimension. For some, the solution is for all work to be carried out in a secure data environment. While this solves one problem, it geographically limits the likelihood of collaboration.

Of further concern is that the confidentiality problem is becoming more complex, especially with regards the recent proliferation of geo-spatially tagged social data. Much of this data from sources such as sensors, check-ins, trip records, and social media can be spatially or aspatially linked to health records, which leads to potential spatial privacy vulnerabilities. The spatial and aspatial linking of geo-spatial social and health data can be done with minimal GIS skills. With mapping API’s such as Google maps becoming more and more user-friendly, a practitioner unaware about spatial privacy can easily map health records that have been linked to geo-spatial social data.

Geoscientists have tackled confidentiality challenges through three main strategies including anonymity, spatial privacy policies, and obfuscation. Among the three strategies spatial data obfuscation or geomasking has generated considerable attention. Novel masking techniques were developed which could be broadly categorized into affine, aggregation, and random perturbation based on the obfuscation strategy employed [7]. While many of these approaches have merit, there remains a disconnect between the concept and real-world utility. Simply put, spatial data sharing, the creation of “safe” maps, and the preservation of confidentiality remains a confusing and often unobtainable task for many health organizations. As a result of this shortfall, and due to facing these types of problems with a local health care system, we conceptualized and then built *Privy*, a utility that can be immediately applied by health organizations based on the principles of *isomasks* [7]. Geocoded health data, such as the addresses of cancer patients, are masked in such a way that the recipient researcher has no information about the original coordinate locations. Yet the spatial configuration of the coordinates is maintained, which is vital for point-based hotspot analyses and even regression approaches (using the attribute columns of the health record as dependent variables). After the spatial science collaborator has performed the analysis, the resulting output can be shared back with the health organization, and can further be re-transformed using a unique set of codes stored from the initial transformation, which allow for the map of results to be overlaid onto the “real” geography. The data providers and the researchers can then discuss the results simultaneously, both viewing the same map output, though with a different geographic underlay.

This paper begins by providing a background on some of the strategies that have been adopted to preserve spatial data confidentiality with a particular focus on geomasking. Next, we discuss the mathematical formulation of point data transformations and re-transformations, and the workflow and technical implementation for *Privy* using some analytical and statistical experiments for illustration. Finally, the paper discusses some of the limitations and shortcomings of *Privy* along with a future direction for this type of spatial data confidentiality research.

## Background

Privacy policies define restrictions for the release of individual location data to third parties [28]. For example, the Health Insurance Portability and Accountability Act (HIPPA) requires health data that are visualized by zip code should have a denominator population of at least 20,000. Besides federal laws such as HIPPA, there are human subject protection procedures implemented by IRBs. Even though IRBs review and monitor the collection and use of personally identifiable information, uncertainty still exists within these bodies regarding what are acceptable risks of disclosure with respect to maps and other spatial outputs [10].

As previously mentioned, the three main spatial privacy preserving strategies include, anonymization, policy-based changes, and data obfuscation or geomasking [6]. Anonymity is mainly concerned with the disassociation of information about an individual, including the location of the individual [25]. One of the commonly used metrics for anonymity is *k*-*anonymity*, which is defined as the imprecision in location information required for making an individual indistinguishable from *k* other individuals [27, 29, 49]. In their seminal paper on k-anonymity, Samarati and Sweeney [44] defined a dataset to be k-anonymous when a combination of values of quasi-identifiers can be indistinctly matched to at least k records. Simply put, a dataset is k-anonymous when every record in the dataset is indistinguishable from k−1 other records. Even though k-anonymity was initially developed to improve confidentiality in typical non-spatial databases, its increasing relevance in spatial data privacy led to development of new methodologies such as spatial k-anonymity [15]. Spatial k-anonymity works by utilizing the underlying population density information to displace confidential point data. Even though spatial k-anonymity has been championed as the most accurate privacy protection measure, its dependency on uncertain and inaccurate data sources such as population density data, makes its practical implementation costly and challenging [53].

Among all spatial privacy-preserving methodologies the most commonly used and studied is spatial data obfuscation or geomasking. Obfuscation can be considered as a combination of statistical and epidemiological techniques to mask location information in a way that can still enable meaningful analysis [7, 25, 52]. The two main goals of spatial data obfuscation are to achieve a balance between personal location information protection, and to extract maximum information from fine scale spatial data [25]. Unfortunately, these two goals are inversely related, i.e. the finer the spatial location involved (often preferred for intervention-style analysis), the greater the risk of re-engineering [36]. Many obfuscation methods such as geomasks [1, 7, 25, 30, 45, 51, 54], grid masks [23], and software agents [32] have been suggested to achieve a balance between confidentiality and data utility.

Geomasks can be generally categorized into affine, aggregation, and random perturbation. Affine geomasks (commonly called isomasks) utilize geometrical translation, rotation, or a combination of both for relocating spatial points. The transformations could be global (where all points are equally transformed), or local (transformations are applied to a small area), based on the scale of implementation. The attractive property of affine transformation is its ability to preserve the spatial structure of the data. This could be particularly advantageous for the subsequent use of spatial analysis or visual exploratory techniques such as clustering. With random perturbation each point in a dataset is translocated by a random distance and angle. While random perturbation is theoretically safe compared to affine transformation, the information loss due to the change in spatial structure is much higher, which limits the ability to use spatial and exploratory analysis. Geomasks can also use a variant of a random perturbation, for example Leitner and Curtis [37] developed the “flipping methodology” which inverts original locations about a horizontal and vertical axis of the map, while Curtis et al. [23] developed a grid-based approach implementing a combination of randomization and Monte-Carlo simulation to assign masked point locations. Clarke [17], in his work on developing a multiscale masking method for spatial point data, utilized digit switching to mask coordinates. In this method, the coordinates are first converted to a Military Grid Reference System (MGRS) which permits encryption at five spatial levels of precision, equivalent to 1, 10, 100, 1000, and 10,000 m. Finally, donut masking [30] extends random perturbation masks by ensuring a user-defined minimum level of geo-privacy. The randomly perturbed points are ensured to be outside of a buffer distance from the original location. For areal aggregation masks, the points are assigned to administrative boundaries such as zip codes, census tracts, and counties and only the aggregated polygons are used for further analysis. Even though such aggregations can preserve spatial confidentiality, the information loss is high and often leads to issues such as the ecological fallacy. The verified neighbor mask [41] utilizes a pool of neighbors for relocating or displacing a spatial point, with all neighbors having an equal probability of becoming the destination location. The advantage of this method is its realistic placement of relocated points, though it is less useful for rural areas. Another recently developed geographic mask is the adaptive areal elimination mask [35], which uses an adaptive filtering technique with aggregated data (for example using census enumerations) to make sure that a minimum population level is reached, before performing the random relocation.

The recent developments in Artificial Intelligence (AI), Internet of Things (IoT), and blockchain have spurred a new wave of interest among researchers to develop novel approaches for preserving confidentiality (both spatial and aspatial). As an example, blockchain technology, which uses encryption and data storage in a decentralized and distributed fashion could be an ideal framework for sharing health data [33]. Apart from storing data in a secure way using encryption, blockchain can be used to create instructions on data ownership and data access (smart contracts [38]) which is particularly useful for tasks such as health supply chain management, data sharing, and consent for clinical trials [33]. One of the recent developments in the area of geospatially-enabled block-chain, FOAM [2], utilizes a crypto-spatial coordinate system for preserving geo-spatial data. FOAM blockchain, apart from validating specific time of an entry, validates the associated proof of location for the entry. Geospatial cryptography [31], which is similar to crypto-spatial coordinate system, utilizes homomorphic cryptography which is defined as a procedure that encrypts data in such a fashion that mathematical operations can be performed on the data without decryption, to securely transfer and analyze geospatial data. Even though nuanced methodologies such as geospatial blockchains are progressing consistently, some of the challenges associated with it such as interoperability, blockchain security, and transparency, still require further attention before full implementation [33]. Software agents provide another methodology for geospatial privacy preservation. This approach is based on controlling access to original individual records without releasing personally identifiable details [32]. Apart from ameliorating the deficiencies presented by releasing spatially aggregated data, the risk of re-identification is much lower with software agents when compared to geo-masked data. Though very promising, the use of software agents to handle confidential health datasets is still at its infancy due to the challenges related to establishing highly secure computer infrastructure. The recent advances in cyberinfrastructure offer promise in the revamping of software agents, though yet again, these methods do not offer immediate solutions to a health care organization requiring spatial expertise *now*.

## Mathematical formulation

### Point data transformation and re-transformation

**Fig. 1:**
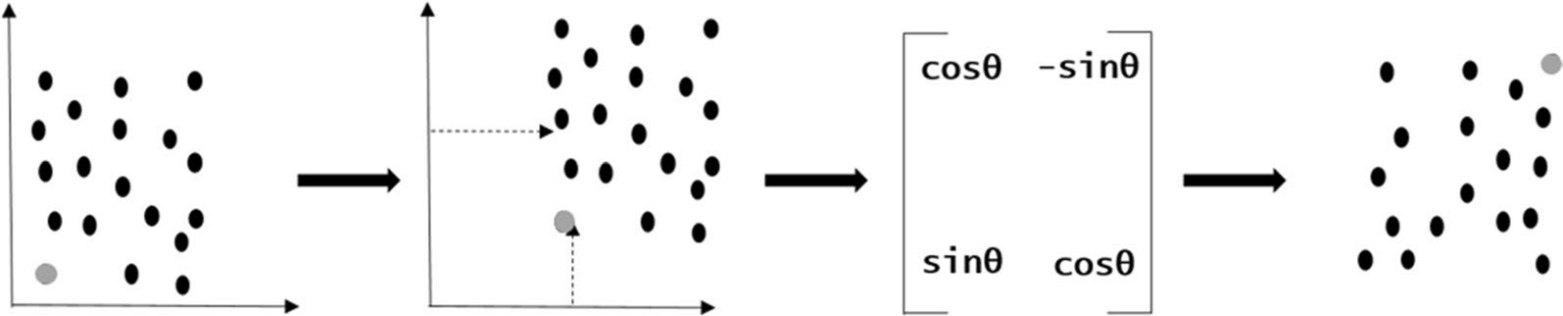
Obfuscation by point data translation and rotation. An offset generated from a random number is used for the translation and the rotation is performed using a rotation matrix. The grey dot indicates a point that has been transformed in space

The *Privy* approach, which belongs to the family of *isomasks* [7], involves a random spatial translation and rotation of an original spatial point dataset. A distance offset is generated from a random number, which is later reused to re-transform the obfuscated data back to the original locations. More specifically, the transformation of the point data involves two steps, a random spatial translation and rotation. For the translation step, an offset is defined to ensure that the newly transformed points exceed a minimum distance from the original point set. This procedure is closely related to donut masking [30], where an inner radius is defined to prevent the transformed points from being accidentally too close to the original points. Suppose the offset intervals are {*X*_*1*_*, X*_*2*_} for x coordinates and {*Y*_*1*_*, Y*_*2*_} for y coordinates, then new coordinates for a location (*x, y*) are displaced at least by (*X*_*1*_–*x*) along the ordinate and (*Y*_*1*_–*y*) along the abscissa. The distance for translation from the original location (*x, y*) is made random by generating a displacement value obtained by multiplying the offset intervals (*X*_*2*_–*X*_*1*_) and (*Y*_*2*_–*Y*_*1*_), with a random number (*r*) (Eq. (1)). As translation maintains the original pattern of the spatial data, the obfuscated points could be potentially vulnerable to identification. In order to tackle this challenge, we perform a random rotation on the translated coordinates. Rotation of coordinates is performed by a matrix multiplication of translated coordinates with a rotation matrix (Fig. 1) which maintains the structural equivalence between the real and transformed coordinates and is essential when re-transforming surfaces generated from the obfuscated spatial data. The random number generated for the translation phase is saved to a local database as a < *key,value *> pair, with the key being a user provided parameter and the random number being the matched corresponding value. Along with the random number, the geographical extent for the transformed points are also saved into the database for a raster re-transformation procedure.

1 $$\begin{aligned} & X = x + \left( {r*\left( {X_{2} - X_{1} } \right)} \right) + X_{1} \\ & Y = y + \left( {r*\left( {Y_{2} - Y_{1} } \right)} \right) + Y_{1} \\ \end{aligned}$$

**Fig. 2:**
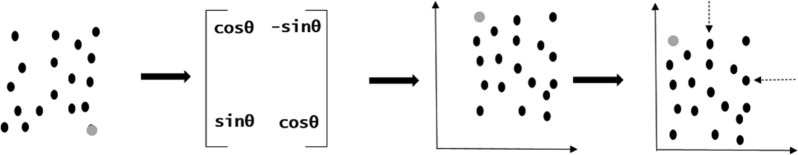
Re-transformation of obfuscated point data through rotation and re-translation. The point data are rotated in space using the rotation matrix and re-translation is performed using the offset generated from the random number. The grey dot indicates a point that has been re-transformed in space

The re-transformation procedure utilizes the random number saved to the local database. First, an X-degree anti-clockwise re-rotation occurs which essentially brings the transformed coordinates into the same orientation as that of the real data. Then the user-supplied key is utilized to retrieve the random number used for the translation, resulting in all coordinates being re-transformed to the original location (Fig. 2) (Eq. (2)).

2 $$\begin{aligned} & x = X - \left( {r*\left( {X_{2} - X_{1} } \right)} \right) - X_{1} \\ & y = Y - \left( {r*\left( {Y_{2} - Y_{1} } \right)} \right) - Y_{1 } \\ \end{aligned}$$

### Raster re-transformation

**Fig. 3:**
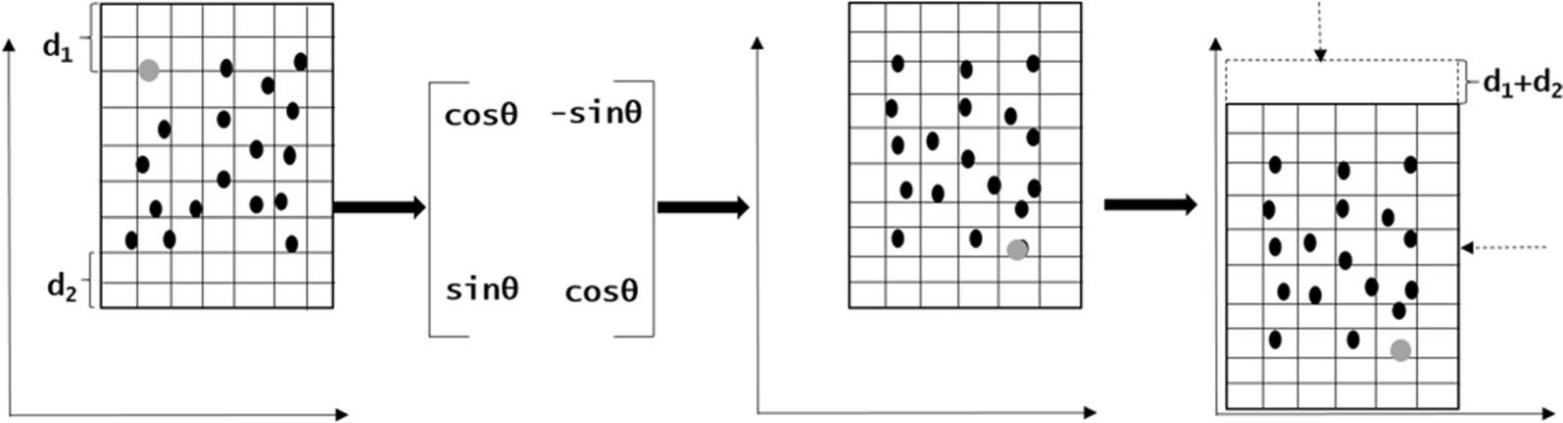
Re-transformation of the raster through rotation and re-translation. The grey dot indicates a point that has been re-transformed in space

While the successful transformation and re-transformation of a point (patient address) data set is a useful academic exercise, the reality behind wanting to perform such a procedure is that outgoing point data will be analyzed by a third party, with (probably) a continuous surface output, most likely a raster image, being returned. For re-transformation of the raster generated from the obfuscated points, the bottom right coordinate of the raster is again rotated X-degree anti-clockwise. This rotated coordinate is the *unadjusted* top left coordinate for the re-transformed raster (*X*_*left*_^*′′′*^*, Y*_*top*_^*′′′*^). A X-degree matrix rotation is then performed to accommodate the data changes due to the orientation of the raster. The re-translation procedure (Eq. (2)) is applied to the *unadjusted* top left coordinate (*X*_*left*_^*′′′*^*, Y*_*top*_^*′′′*^) of the re-transformed raster using the random number used in the obfuscation (again retrieved from the local database). Even though the raster has been transformed into the original space, an alignment issue due to the rotation of points needs to be addressed (Fig. 3). The spatial extent of the obfuscated points (*X*_*left*_^*′*^*, Y*_*bottom*_^*′*^*, X*_*right*_^*′*^*, Y*_*top*_^*′*^) retrieved from the local database and the spatial extent of the raster created from the obfuscated points (*X*_*left*_^*′′*^*, Y*_*bottom*_^*′′*^*, X*_*right*_^*′′*^*, Y*_*top*_^*′′*^) can be used to calculate the *adjusted* top left coordinate for the re-transformed raster (*X*_*left*_*, Y*_*top*_). At first, the difference in spatial extent for the top, bottom, left, and right of the obfuscated point data to the corresponding raster generated from the obfuscated data are calculated (*x*_*ldiff*_*, x*_*rdiff*_*, y*_*tdiff*_*, y*_*bdiff*_) (Eq. (3)).

3 $$\begin{aligned} & x_{ldiff} = X_{left}^{'} - X_{left}^{''} \\ & x_{rdiff} = X_{right}^{'} - X_{right}^{''} \\ & y_{tdiff} = Y_{top}^{'} - Y_{top}^{''} \\ & y_{bdiff} = Y_{bottom}^{'} - Y_{bottom}^{''} \\ \end{aligned}$$

The differences for the top and bottom as well as the left and right are added to calculate the adjusted values (*x*_*adj*_*, y*_*adj*_) (Eq. (4)).

4 $$\begin{aligned} & x_{adj} = x_{ldiff} + x_{rdiff} \\ & y_{adj} = y_{tdiff} + y_{bdiff} \\ \end{aligned}$$

Based on Eq. (5), the final adjusted top left coordinate for the re-transformed raster (*X*_*left*_*, Y*_*top*_) can be calculated.

5 $$\begin{aligned} & X_{left} = X_{left}^{'''} - x_{adj} \\ & Y_{top} = \left\{ {\begin{array}{*{20}l} {Y_{top}^{'''} + y_{adj} , y_{adj} < 0 } \hfill \\ {Y_{top}^{'''} - y_{adj} , y_{adj} \ge 0} \hfill \\ \end{array} } \right. \\ \end{aligned}$$

## Workflow and technical implementation

**Fig. 4:**
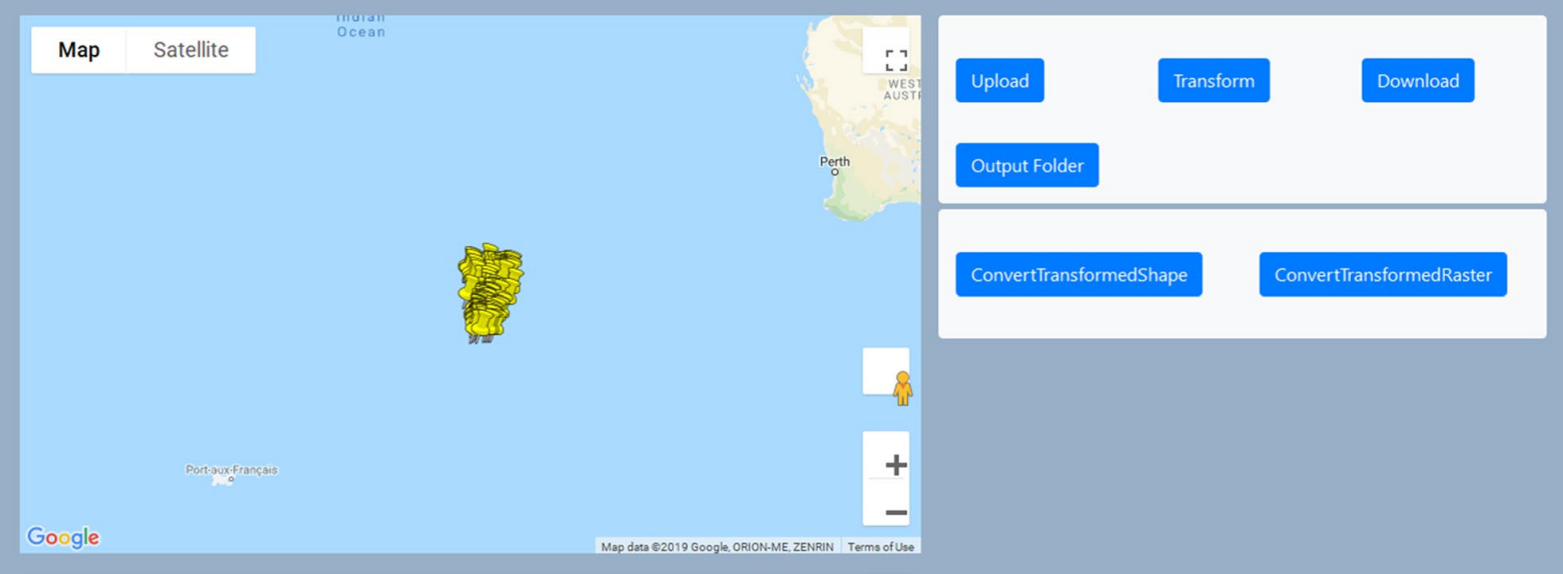
User interface for *Privy*

Unlike with other academic approaches to obfuscate data, *Privy* was conceptualized while simultaneously being developed as a ubiquitous tool. This is important to emphasize in that the driving factor behind developing *Privy* was that it could immediately serve as a health organization/spatial science collaborating framework. To achieve this goal, a simple user-friendly interface was developed using Html5, and JavaScript (Fig. 4), while Google Maps API, which is a JavaScript based map framework from Google, was used to visualize the obfuscated data. All the algorithms for obfuscation and re-transformation were written in Python, and complex operations such as the matrix rotation was done utilizing the mathematical library Numpy. SQLite3 was used for saving parameters such as the random values and the extent of the transformed coordinates. PyQT, which is a Python framework with an in-built browser that could support both web components and Python based core components was used to connect the web-interface with the local database.

**Fig. 5:**
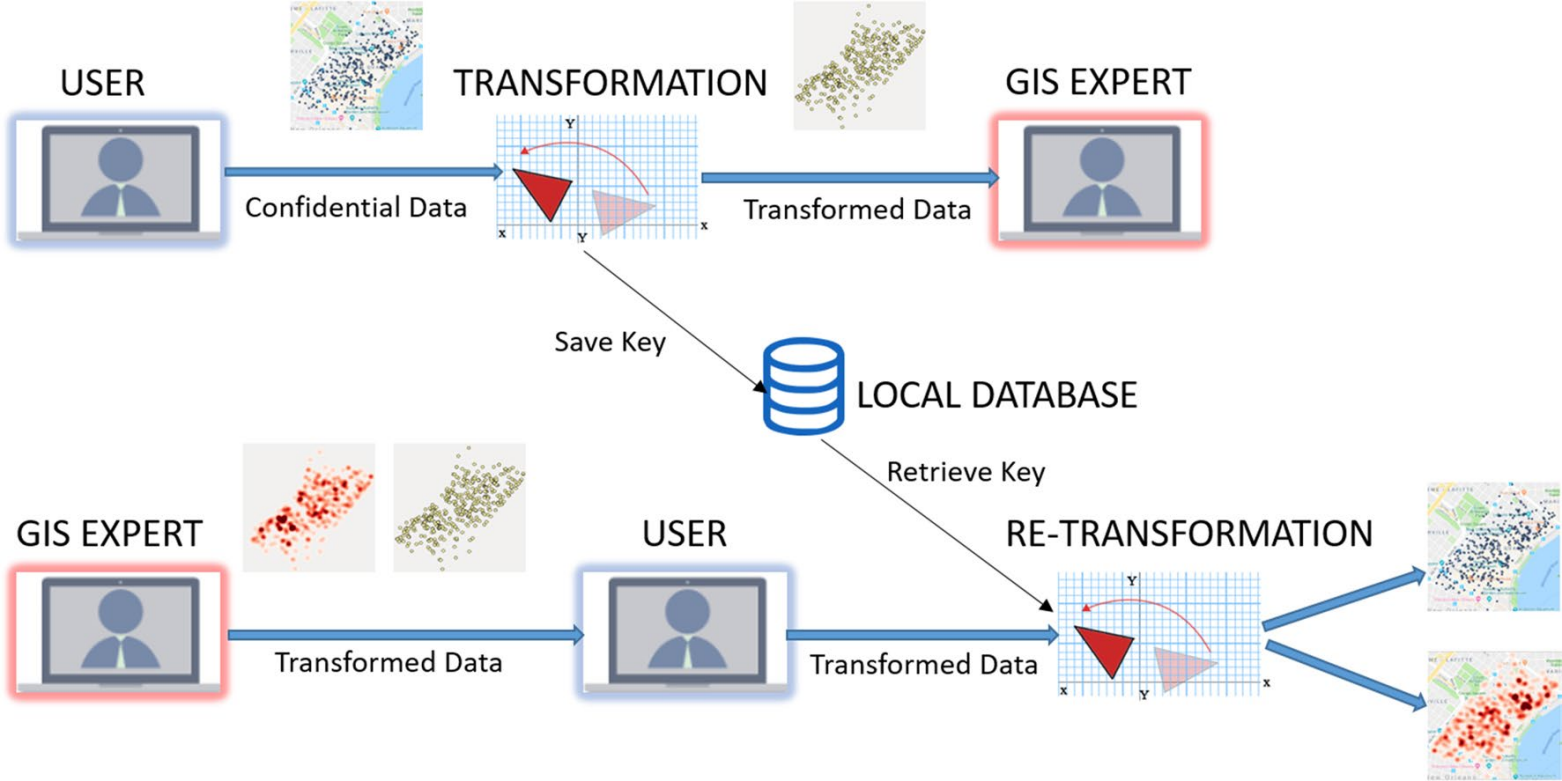
*Privy* workflow for data obfuscation and re-transformation

As a first step in the coordinate transformation, confidential point data, such as patient addresses are uploaded as an ESRI shapefile. These data are then transformed as previously described using *Privy*, with the new data also being output as a shapefile. The transformation key is stored for use on the eventual re-transformation, and the health organization waits for its collaborator to perform an analysis and return the output. A re-creation of the obfuscation procedure occurs with the returned analytical output and both parties can then interpret the findings on the same output map, though overlaid on a different Geography (Fig. 5).

## Experiments

**Fig. 6:**
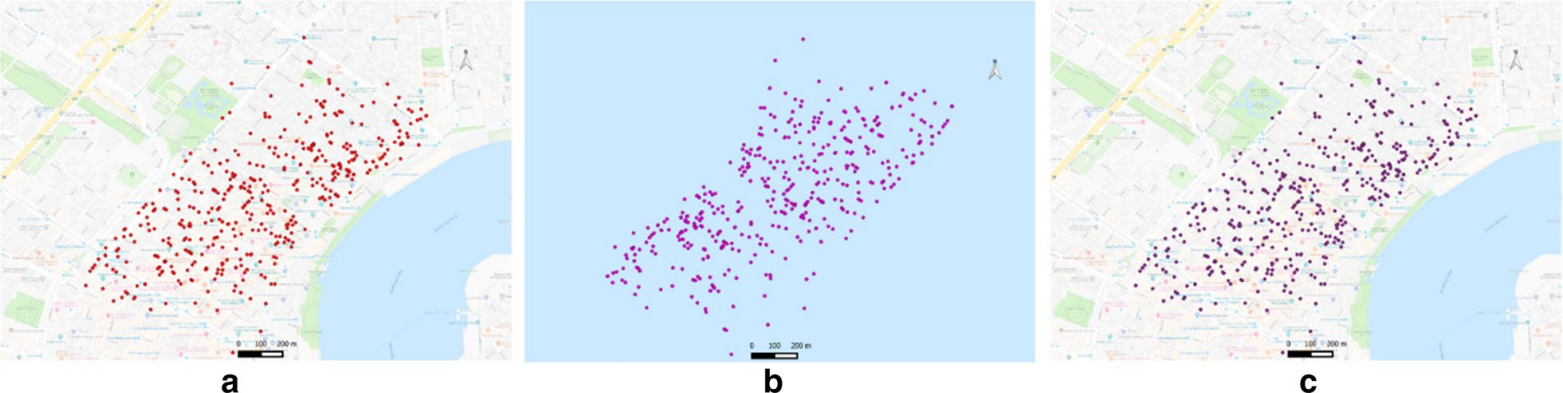
**a** Unmasked yellow fever death data, **b** obfuscated data, **c** re-transformed data

In order to show the utility and effectiveness of *Privy* as a methodological approach that could act as a conduit between health data guardians and collaborating researchers, a series of experiments were conducted. In order to test whether *Privy* was able to obfuscate and correctly re-transform spatial point data, the 1878 yellow fever epidemic of New Orleans, Louisiana [21, 24] was utilized. This dataset, using mortality locations recorded in the *Official Report of the Deaths from Yellow Fever as Reported by the New Orleans Board of Health* (1879), illustrates a more typical health application as the age, date of death, and nativity, are linked to a residential address. Indeed, it has previously been suggested that these data provide an excellent test set for confidentiality work as they are at address level, are “real” epidemic data, but there is no consequence in terms of a real-world reengineering risk [21, 24]. The case locations were obfuscated using *Privy*, and then re-transformed back into the original space for comparison. In order to test the correctness of the re-transformation procedure, a custom Python script was used to calculate the point-by-point distance comparison between the original and re-transformed dataset. The point data maps (Fig. 6) shows the real location of yellow fever deaths (Fig. 6a), the obfuscated locations (Fig. 6b), and the re-transformed locations (Fig. 6c) respectively. By visual examination alone, we can see that the re-transformed locations and the real locations are similar. The unique ids for each coordinate are used to facilitate a one on one comparison with the real and re-transformed data. The output of the point-to-point distance calculation for each pair of coordinates is zero, which indicates an exact re-transformation of the obfuscated spatial dataset (Fig. 6b).

To test the impact of obfuscation on the spatial structure of point data, a set of spatial point pattern analyses were conducted, though for the sake of brevity only two experiments are described here. The Average Nearest Neighbor, a common clustering technique for point data [50] was run on both the real and obfuscated yellow fever datasets. Euclidean distance was used as the distance relationship between the point data. The results reveal clustering (*nearest neighbor ratio *=* 0.621659*) with statistical significance (*p value *=* 0.00*) (Table 1).

**Table 1:** Average nearest neighbor results for yellow fever unmasked and obfuscated data

	OMD	EMD	NNR	z-score	p-value
Real	19.24	30.96	0.62	− 16.23	0.00
Obfuscated	19.24	30.96	0.62	− 16.23	0.00

*OMD* represents observed mean distance, *EMD* represents expected mean distance and *NNR* represent nearest neighbor ratio

For the second experiment, Ripley’s K function [42], a technique used to investigate clustering, was applied to both the real and obfuscated yellow fever dataset. The results (Table 2) reveal a high level of clustering for small distance bands and a subsequent reduction in clustering at higher distances. The difference value for observed (L(d) transform) and expected (distance of band itself) values, Diff, increases up to band four (188.6 m), and further decreases till band ten (472.15 m). A comparison of transformed values and differences for masked and unmasked data reveals exact matches for all distance bands.

**Table 2:** Ripley’s-K function results for unmasked and obfuscated data

Distance	L(d)_R_	L(d)_O_	Diff_R_	Diff_O_
47.21	73.24	73.24	26.02	26.02
94.43	124.45	124.45	30.02	30.02
141.64	176.63	176.63	34.99	34.99
188.86	227.46	227.46	38.61	38.60
236.07	273.51	273.51	37.44	37.44
283.29	318.81	318.81	35.52	35.52
330.50	359.59	359.59	29.08	29.08
377.72	398.78	398.78	21.06	21.06
424.93	437.85	437.85	12.91	12.91
472.15	473.88	473.88	1.73	1.73

*L(d)* represents transform value and *Diff* represents the difference between the expected and observed value. The subscripts R and O represents real and obfuscated results

**Fig. 7:**
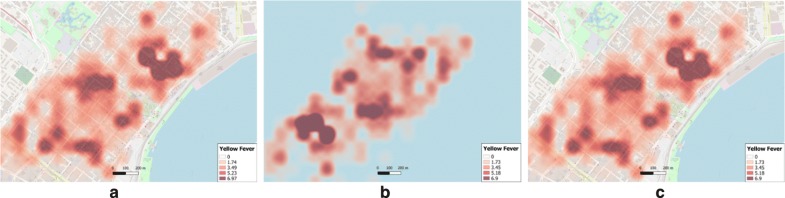
KDE surfaces generated from **a** original yellow fever data, **b** obfuscated data and **c** re-transformed raster

The results of the spatial analysis indicate that *Privy* preserves spatial structure during data obfuscation and is able to successfully re-create the original results. To further analyse the capabilities of *Privy*, a set of surface generating experiments were conducted. Many of the visual exploratory and interpolation techniques in GIScience such as Kernel density estimate (KDE) [47] and Inverse distance weighted interpolation (IDW) [39], generate raster surfaces from a set of spatial points, and as such both were utilized here. Initially, the surfaces were generated from the original yellow fever data. Then, *Privy* was used to obfuscate the original data and the two methods were again applied on the obfuscated data. The raster surface generated from the obfuscated data was re-transformed using *Privy*. Comparison occurs by spatially matching the spatial coordinates of the raster extent, the cell size, and total rows and columns. The KDE results for the unmasked (Fig. 7a) data raster shows multiple hotspots with a major focus in the north eastern sector. The obfuscated data raster (Fig. 7b) shows an inverted pattern but with similar values in the transformed space. The re-transformed data raster (Fig. 7c) reveals the same trends as in the real data raster (Fig. 7a).

**Fig. 8:**
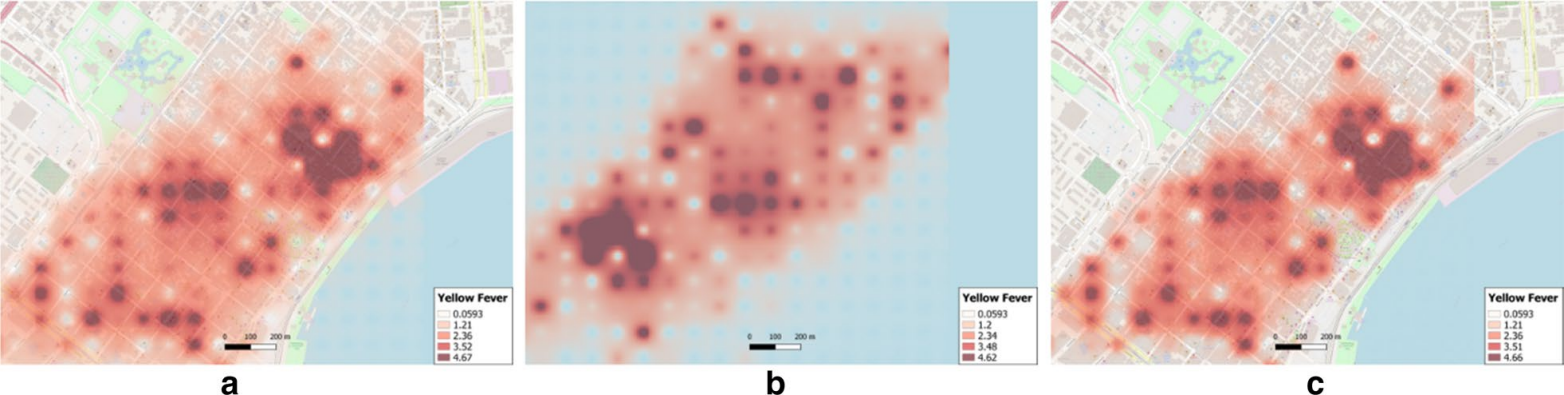
IDW surfaces generated from **a** original yellow fever data, **b** obfuscated data and **c** re-transformed raster

The IDW results for the unmasked data (Fig. 8a) also reveals relatively high values for interpolated yellow fever in the same location. For the obfuscated data, the IDW results (Fig. 8b) indicate an exact inverted pattern of the unmasked data (Fig. 8a). The re-transformed raster (Fig. 8c) shows the exact same pattern as the raster generated from the unmasked data (Fig. 8a).

## Discussion and conclusions

There is an immediate need for health organizations and skilled geospatial researchers to collaborate on various health concerns. Simply put, understanding fine scale processes in outcomes such as asthma, infant mortality or overdoses, can lead to changes in intervention. The challenge is how to share data, and perform fine scale spatial analyses safely, where there is an extremely limited likelihood of a confidentiality violation. While making data available at coarser aggregations such as census tracts or zip codes might satisfy the creation of health atlases or public presentations, intervention strategies require finer scale spatial data. Therein lies the spatial-confidentiality dilemma—the data guardian must balance the increased risk of using/releasing fine scale data with the potential improvements in health.

While researchers have considered this dilemma conceptually for decades, arguably the debate has shifted as health organizations become more spatially literate; more clinicians and other health researchers now are aware of the power of mapping and how it might advance current thinking, especially with regards more effective targeting of intervention. In other words, we have moved from largely academic discourse to a real-world need. A solution to bridge the health and spatial research sectors are required as many organizations (health departments, hospitals) have limited or no GIS expertise. Even if such skill lies within a department of an organization, the siloed nature of health research treats each unit as though they are outsiders. It is almost as hard for a geoscientist working in a diabetes unit to offer spatial research help to a cancer centre, even within the same hospital. As a result only basic mapping, or worse, incorrectly run and interpreted spatial analyses often occur. One solution is to obfuscate data in such a way that collaborative teams can work together, in near real time, without running the risk of violating patient confidentiality.

While there have been many eloquent approaches to solve this problem, these have largely remained in the realm of academia. If a hospital wants to share data with a collaborator, there is no widely adopted solution, especially one that can be applied with a limited geospatial skillset by a healthcare analyst. In this paper we addressed this problem using a three pronged approach; design a method that was simple to understand, that was powerful in both protecting confidentiality and allows for a variety of different analytical approaches, and that could be applied now, in any health organization with just a minimum of spatial data understanding.

We have achieved this with *Privy*. Our results show that the obfuscation technique applied to point level data preserves spatial structure, which in turn provides the exact same results for masked and real data, achieving one of the overarching goals of geomasking [25]. Future comparative analyses should incorporate other techniques important to health research, such as SaTScan or LISA [4], though we have no reason to believe these results will be any different.

The ability to re-transform surfaces generated using obfuscated data to its original location adds further potential to this approach. This is important both in terms of being able to share output and have a simultaneous interpretation between both parties, and even being able to share finely aggregated original surfaces without concern. Even though KDE continuous surfaces are less prone to confidentiality issues, bullseye effects in remote areas still run an unacceptable risk of re-engineering [10]. The obfuscation of the raster surface as displayed in this paper provides a solution to this vulnerability of isolation.

While this approach is available now, some limitations need to be addressed. Firstly, the current approach requires address level data to be geocoded, and output as a shapefile. While this might be a limitation for some organizations, some electronic medical record systems now offer geocoding as output, and the basic use of a GIS’s functionality is becoming more commonplace. Even so, for full ubiquitous use, for example with a small county health department or hospital, a pre-module that provides geocoding services and shapefile creation would be a useful evolution.

Secondly, the only data that can be shared has to come from the health organization (or a similar unit). Publicly available data layers like boundaries, street files, or census data cannot be shared as this increases the risk of re-engineering. While this may limit the use of some techniques, such as regression, more and more socioeconomic, behavioral and even environmental data are being collected by health organizations. These could provide a set of independent variables linked to the original patient file as attributes. With these added then the comparison of real and obfuscated data based on spatial modelling techniques such as ordinary least squared regression (OLS) and geographically weighted regression (GWR) could be further explored. Indeed, one spill over benefit with the availability of tools like *Privy* is a greater incentive for the recording of more data *inhouse*, while making temporal changes (both biological and address related) more easily accessible for spatio-temporal analysis. In future revisions of Privy we plan to incorporate secured spatial joins and aggregations, which could be particularly useful for incorporating external datasets. Along with providing aggregated results, It would also be beneficial if Privy could automatically identify and warn the user about potential vulnerabilities such as a lack of a substantial denominator within the analysed data (addressing the previously mentioned bulls-eye effects).

Finally, the main vulnerability of the *Privy* approach is if a bad actor has information about one patient, then conceptually it is possible that this address could be used to re-engineer the rest of the system. While this will always be possible, it is unlikely given that the required data would have to have the exact input of the data being transformed. It is not enough to know a birth weight, or a BMI, or a blood lead level count as these are likely to be replicated across the data set, and for many these also vary with medical visit. Therefore, the bad actor would have to have access to the electronic medical record file of one person, and then be able to place that within the transformed and rotated data. This is even more unlikely if the geospatial team does not know which city the original data come from. Finally, the standalone nature of the software and the local database, add a further layer of security as the key used for masking and re-transformation are only available with the health organization.

In summary, as custodians of medical and health data records often have minimal GIS expertise, it is essential to develop simple yet efficient software methodologies to help them preserve spatial confidential and at the same time enable collaborative research with GIS experts. We have achieved this by developing the *Privy* technique, a tool which is already being used in real-world situations to address the spatial confidentiality dilemma.

## Data Availability

Source code for Privy and the Yellow Fever Dataset available in https://github.com/ghhlab/confidentiality.
